# 

**Published:** 1881-04

**Authors:** 


Vol. XVII. April 1881. No. 1.

### The Bistoury.

Thad. S. Up de Graff, M.D.

EDITOR.

ELMIRA, N.Y.

